# Missed opportunities for improving practice performance in adult immunizations: a meta-narrative review of the literature

**DOI:** 10.1186/s12875-017-0694-1

**Published:** 2017-12-22

**Authors:** Natalia Loskutova, Craig Smail, Brian Webster, Kemi Ajayi, Julie Wood, Jennifer Carroll

**Affiliations:** 1 American Academy of Family Physicians National Research Network, 11400 Tomahawk Creek Pkwy, Leawood, KS 66211 USA; 2 Wilmington Health, 1202 Medical Center Drive, Wilmington, NC 28401 USA; 3 Department of Family Medicine, University of Colorado School of Medicine, 12631 E. 17th Ave, Aurora, CO 80045 USA

**Keywords:** Missed opportunity, Meta-narrative literature review, Adult immunizations

## Abstract

**Background:**

We sought to characterize how the term “missed opportunities” is reported in the literature in the context of immunization rates and to assess how missed opportunities can be operationalized.

**Methods:**

Peer-reviewed literature searches were conducted in April – May, 2015, to answer: “What methods research studies used to operationalize *missed opportunities to vaccinate*?” A meta-narrative review methodology was used.

**Results:**

Seven studies met inclusion criteria. The methodologies for quantifying missed opportunities fell into two general categories based on: 1. the number of healthcare encounters per patient without appropriate vaccination services, defined as a number of visits per patient with no vaccination related services (Missed opportunities per patient); 2. vaccination status as “non-vaccinated” among a group of patients who had a healthcare encounter where the vaccination should/could have had happened (Missed opportunities per population).

**Conclusions:**

Our study provided an initial overview of the methods reported in the literature, and concluded that the quantifiable *missed opportunity* holds promise as a measurable outcome (variable) for research and quality improvement projects aimed to increase adult immunization recommendation and uptake in primary care.

**Electronic supplementary material:**

The online version of this article (10.1186/s12875-017-0694-1) contains supplementary material, which is available to authorized users.

## Background

Developing and providing immunizations are two of the greatest public health achievements. Adult immunization rates in US, however, remain well below *Healthy People 2020* suggested targets for influenza vaccinations in adults >65 (target 90%), and for pneumococcal disease in adults ≥65 years (target 90%), and in persons at high risk for pneumococcal disease aged 18–64 (target 60%) [1]. Despite scientific advances in vaccine development, morbidity and mortality from preventable infectious diseases persist due to multiple documented barriers. Each year US primary care physicians provide more than 560 million office visits and are in the unique position to administer immunizations to patients of all ages [2]. However, health care encounters in which a person eligible to receive vaccinations is not vaccinated [3] occur in any setting where vaccinations are offered, and these occasions are viewed as missed opportunities to provide adequate vaccination for older adults [4].

It is common in published literature on vaccination to state that missed opportunities to vaccinate exist and likely contribute to the persistently high levels of vaccine-preventable illness and gaps in achieving the population-wide Healthy People 2020 goals. In published literature, however, missed opportunities to immunize are often referred to as an abstract concept that represents the end-result of a patient not being vaccinated due to existing barriers ranging from patient resistance, provider bias, or vaccine unavailability. The term *missed opportunities* generally is not characterized well and is often used interchangeably with other terms that refer to poor immunization status in individual patients or populations.

Scientific evidence suggests that in order to assess and potentially change a measure, that measure or term needs to be measurable and operationalized. In the social sciences, operationalization is commonly referred to as the process through which abstract concepts are translated into measurable variables [5]. This suggests that *missed opportunities* could be potentially developed into a measure of immunizations. However, it is unknown if *missed opportunities* can be systematically measured in a meaningful and repeatable way. In order to address missed opportunities, there is a need to define, quantify, and track missed opportunities. From a population health perspective, operationalizing *missed opportunities* for immunizations could allow us to identify strategies to reduce the proportion of primary care visits that are viewed as missed opportunities to vaccinate and subsequently to close the gap on vaccine-preventable illness.

The overall goal of this work was to characterize how the term *missed opportunities* is reported in the literature in the context of immunization rates and to assess whether and how *missed opportunities* can be operationalized. Specifically, the objectives of this paper are to: 1. characterize the missed opportunities in adult vaccinations in published articles relevant to primary care; 2. synthesize evidence pertaining to operationalization of missed opportunities concept; and 3. explore the potential of operationalized or quantifiable missed opportunities as a measurable outcome (variable) for research and quality improvement projects focused on adult immunizations in primary care.

## Methods

### Study overview

This is a meta-narrative review study. We selected this method because we sought to assess and synthesize published evidence around operational definitions of missed opportunities for adult vaccinations for which, to the best of our knowledge, no operational definitions currently exist. The meta-narrative review methodology was designed specifically to summarize a research topic with no standard conceptualization (i.e., researchers bring different presumptions and goals to the research) and different paradigms (i.e., potential to explore more complex and diverse questions in one review, the possibility to include studies of different designs, utilizing multiple outcomes, varying systems, and different methodologies). Additionally, the meta-narrative review typically includes two key components: exploration of literature and the synthesis of data, resulting in the meta-narrative maps of the findings that highlight differences and similarities of various paradigms. In contrast, a systematic literature review is designed to summarize and ultimately grade the quality of evidence and the results of available carefully designed clinical trials to evaluate the evidence on the efficacy or effectiveness of healthcare interventions. Since the focus of this review was not on grading the quality of evidence related to immunization interventions but on the methodology for operationalizing a new outcome, the meta-narrative review methodology is a better fit for the objectives of the study. Thus, the meta-narrative review method was selected primarily due to the purpose of the study, the lack of standard definition of missed opportunities in the field. and the above-mentioned characteristics of the meta-narrative review methodology. We followed the meta-narrative review framework proposed by Wong, et al. [6]. We conducted a comprehensive meta-narrative review of the biomedical literature identified in the PubMed database that was published between January, 2000, and May, 2015.

### Literature searches

We included studies of any design (i.e., randomized, observational, cross sectional, descriptive, qualitative), size or setting published in English in which adult immunizations was the primary outcome. We also included non-original or summarized literature (i.e., systematic or non-systematic literature reviews, commentaries).

A professional librarian employed full-time at the AAFP Leawood, KS, office, completed the initial literature search in April – May, 2015. To start the search, the following key words were used: immunizations; missed opportunities; vaccination; immunization schedule; and physician practice patterns.

The overall eligibility criteria were as follows:

- the study cohort consisted of adults 18 and older
- the focus of the study was on adult immunizations
- the study was completed in 2000 or later
- the study was published in English

Since the term *missed opportunities* is not a term in the MeSH controlled vocabulary (and hence is not included as a PubMed keyword), we searched the term in both the title and abstract of indexed articles. We also searched with conceptually related MeSH key words. From the results of both approaches, we identified records for title and abstract screening and review. See Additional file 1 for the full search strategy.

### Meta-narrative review process

To characterize missed opportunities for adult immunizations, we first reviewed the titles and abstracts of papers retrieved using the aforementioned search criteria and key words. The titles and abstracts of all identified articles were reviewed by two reviewers (NL and CS) and selected for full text review based on the following: the articles should focus on immunizations in adults; the methods and/or results section of the abstracts should include evidence of or indication of use of data for missed opportunities; and/or describe methodology for how the term was operationalized or quantified; and/or have a numerical exemplification of missed opportunities presented in results.

Next, the records were included in the further full text review if they were deemed relevant to the objectives of the study based on full or partial alignment with the inclusion criteria. For those records, full text copies were obtained and included in full review. The articles were excluded if they did not provide a specific definition for missed opportunities or relevant methodology or did not include quantifiable results for missed opportunity in the abstract.

Three individuals (NL, CS, JW) reviewed the full text articles; at least two individuals reviewed each article. Full texts of these articles were reviewed using a template to summarize the study as follows: name (title, authors, date); purpose; population and setting; service or procedure; timeframe; methodology; missed opportunity definition; significant results or observations; methodological advantages and limitations. The articles were included in the results if they had a clear definition of missed opportunities, quantified or operationalized missed opportunities according to their definition or formula, and included a corresponding quantitative measure of missed opportunities in outcomes using data. The articles that originally partially aligned with the guiding review principals that did not provide additional information to satisfy all requirements including a specific definition for missed opportunities, relevant methodology or did not include quantifiable results for missed opportunity in the full text were excluded. Additionally, we reviewed reference lists from eligible studies and conducted additional searches for publications that cited eligible studies. The full texts of these articles were reviewed following the same process as above. The literature search and data extraction were completed in September, 2015.

## Results

### Literature search and selection results

**Fig. 1:**
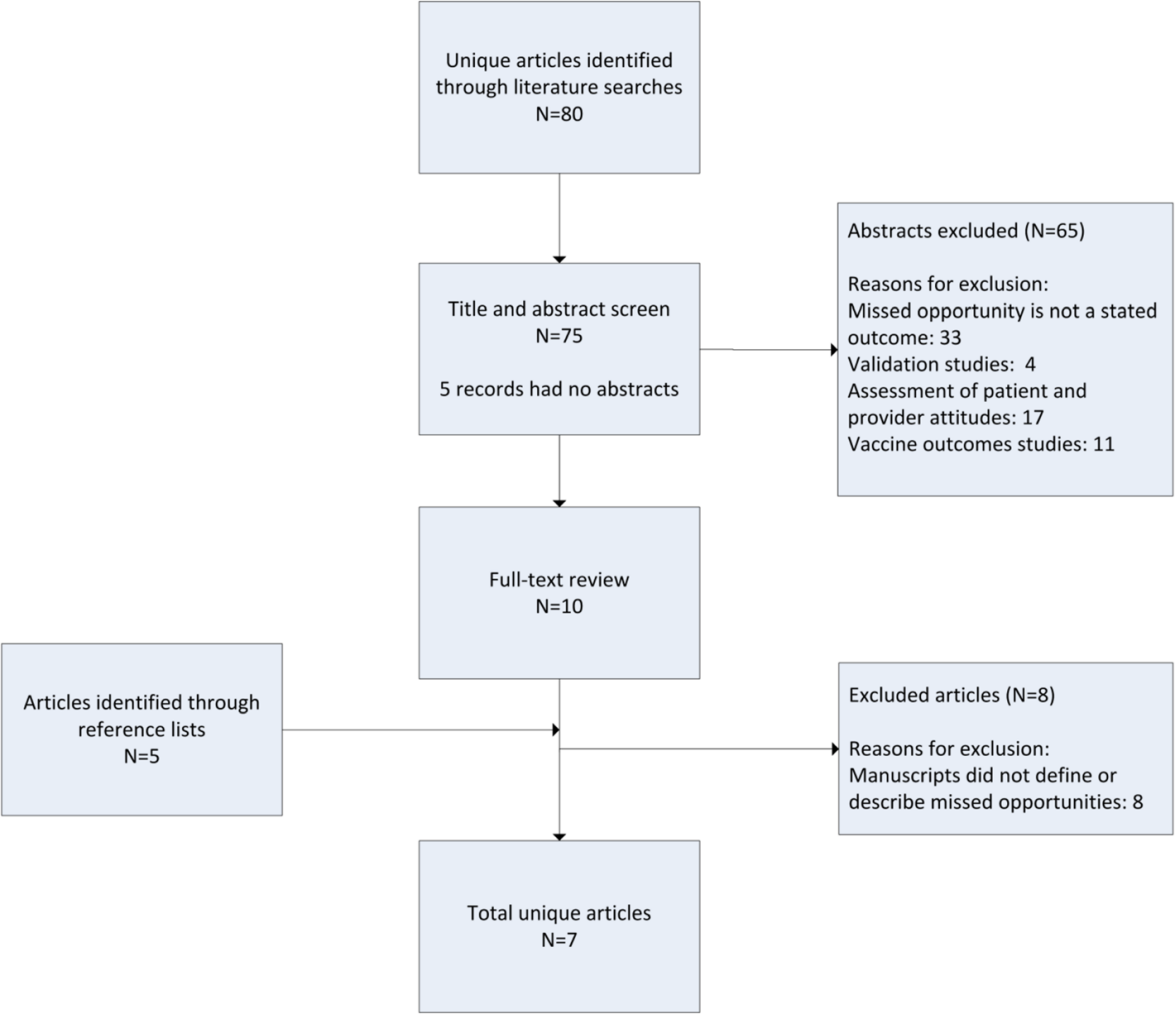
Article selection process

The literature search for the systematic review identified 80 possibly relevant citations. We included seven studies. The study selection process is described in Fig. 1.

### Overview

A total of seven articles were included in the final sample. Of the total, two studies focused on influenza vaccinations only, four studies focused on pneumococcal and influenza vaccinations and one study included influenza, pneumococcal and tetanus adult vaccinations. The studies enrolled a combined total of 15,140 participants. The patient populations for the majority of the studies were individuals aged 65 years or older. The patients studied were from a variety of backgrounds including health centers in disadvantaged neighborhoods, Veterans Administration clinics, rural and urban ambulatory practices and hospitals. All studies utilized observational designs - four studies extracted data from retrospective reviews of medical records, one study had direct observations at the time of visit, and three studies incorporated surveys or interview data. Of the total, three studies were conducted in inpatient hospital settings and two were conducted in the ambulatory primary care. The summary of studies is presented in Table 1.

**Table 1:** Descriptive table summary of the studies that used data derived missed opportunity for clinical or preventative service or procedure. Observational, review and interventional studies in any clinical topic area are included

Study purpose; Population and setting; Service or procedure; Timeframe; Location	Methodology (study design, health IT use, how the data were obtained)	Missed opportunity definition (data elements used; formula or other for deriving the value for missed opportunities)	Significant results or observations	Methodological advantages or limitations
Nowalk, et al., 2004 [8]				
Purpose: To identify missed opportunities and find documentation of immunizations in medical records. 810 participants age ≥ 66 years are enrolled from 3 health centers located in disadvantaged neighborhoods. Sites include 3 VA clinics; 8 rural practices; 9 rural/urban clinics, all belonging to the same health network. Service: Adult immunizations (influenza, pneumococcal, tetanus) Review of visit data: 1998–2000 for influenza; 1995–2000 for PPV and tetanus Location: USA	*Retrospective* review of paper medical records. Exclusion criteria for review: patient data were excluded if the date of the first visit was after the recorded date of the vaccine receipt or if the patient was not seen during the review period (i.e., 1998–2000 for influenza; or 1995–2000 for PPV/tetanus)	For Tetanus and PPV: Administered to all patients without recorded vaccination: missed opportunities = total visits minus any visits in which vaccine was discussed or refused. For Influenza: visits during October–February where influenza vaccine was not given, discussed or refused.	Immunization rates were 24.1% for annual influenza, 49.1% for pneumococcal polysaccharide and 28.6% for tetanus vaccine. During the 27-month study period, patients averaged 1.3 +/− 1.9 acute visits, 6.9 +/− 5.1 chronic visits and 0.48 +/− 0.91 preventive visits (mean +/− S.D.). Missed opportunities to vaccinate ranged from 38.4 to 94.5% of visits.	Advantages: elucidated disparities in rates of missed opportunity amongst clinic type and setting (e.g., disadvantaged urban vs. suburban); good description of how missed opportunity were determined based on the visit and its outcomes. Limitations: retrospective medical record review period for influenza was shorter than other vaccinations - limits comparisons; study was conducted before electronic medical records were generally available
Singleton, et al., 2005 [11]				
Purpose: To assess influenza and pneumococcal immunization rates (and racial disparities thereof) by telephone interview of adults age 65+. 1839 adult respondents (age 65+) completed a telephone questionnaire. Service: Influenza and pneumococcal vaccinations Timeframe for Influenza: 2002–2003 season; timeframe for pneumococcal questions: “recently, or ever, vaccinated” Location: USA	Interview of respondents reporting past vaccinations via telephone	Missed opportunity = had doctors visit, but did not receive a flu shot or a recent recommendation for one Data used to classify M missed opportunity O: clinic visit dates, vaccination status, physician recommendations for vaccination, knowledge of vaccine recommendations	67.8% had influenza vaccine in 2002–03 season; 60% had ever received pneumococcal (range 44% - 63%, depending on race, which was a significant predictor of vaccination status). Missed opportunity rates (recent doctor visit and vaccine recommendation from provider, but no vaccine): blacks, 26.9% versus 7.9%; Hispanics, 19.9% versus 12.1%; and white non-Hispanics, 16.2% versus 6.1%.	Advantages: good geographic spread of respondents (i.e., sampled from across USA); collected data on reasons for vaccination refusal; the results were concordant with those of other national surveys Disadvantages: study relied on patient-reported data; known confusion with pneumococcal vaccination among respondents; did not directly measure actual opportunities for, or refusals of, vaccination; recommendation date is necessarily limited to what the patient remembers/were aware of (e.g., doctors may have neglected/chose not to share recommendation with patient)
Weightman, et al., 2003 [13]				
Purpose: To determine number of missed opportunities 101 patients ages 3 months - 97 years (mean = 63.5), enrolled after being admitted to hospital with a pneumococcal infection. Service: Pneumococcal vaccination Timeframe: 1990–1999 Location: UK	Retrospective manual review of hospital records; consultation with patients’ primary care providers; archived notes (deceased patients only)	Surveyed patients admitted to hospital with pneumococcal infection. If it was found that a patient was not vaccinated, investigators followed up with their primary care physician (and hospital encounters, if applicable) to determine number of missed opportunities (opportunity for vaccination defined as: in the 5 years preceding hospital admittance, patient had one or more of: family physician consult; attended hospital outpatient department; have been admitted to hospital).	Vaccination rate of 5% in those where an opportunity existed Missed opportunity: 30/101 (29.7%)	Disadvantages: patients resided in UK (likely have different guidelines); small sample size (*n* = 101)
Kyaw, et al., 2006 [9]				
Purpose: To characterize vaccination status and opportunities for vaccination patients who had been hospitalized with invasive pneumococcal disease: Adults age 18+ with invasive pneumococcal disease (*n* = 1878) Pneumococcal vaccination Timeframe: 2001–2003 Location: USA	Retrospective chart review	Missed opportunity definition: ≥1 healthcare encounter (including hospitalization, ER visit, outpatient visit) in the 2 years prior to pneumococcal infection	Of 617 unvaccinated patients who were eligible for a vaccination, 566 had at least one opportunity for vaccination; during 1 year Missed opportunity =92%	Disadvantages: not limited to opportunities for vaccination at primary care setting (also included cardiologists, endocrinologists, other specialties)
Skull, et al., 2007 [12]				
Purpose: To examine missed opportunities for recommended influenza and pneumococcal vaccines among hospitalized elderly patients 4772 hospitalized patients 65 and older with pneumonia vaccinations and risk factor assessment Timeframe: April 2000–March 2002 Location: Australia	Self-reported survey of previous hospitalizations and number of doctor visits and provider-confirmed vaccination records	Missed opportunity = provider-subject encounter: doctor visit in the year before hospitalization (or a number of visits, each is a missed opportunity) and/or a hospitalization to the same hospital in the past 5 years	Mean estimate of visits is 11.7 per year (range 0–20); 99.8% of unvaccinated patients had at least one missed opportunity within a year. Influenza – 99.6% missed opportunity with at least one visit PPV – 99.8% missed opportunity with at least one visit in 1 year for flu and 5 years for PPV	The study was conducted in a hospital in Australia; only patients with pneumonia were included; encounters are self-reported on a range scale with increment of 5 (0–4, 5–19, 10–14, 15–19, 20 and more) for last year and yes/no for 5 previous years; automated prompts for providers are recommended as a potential solution in discussion.
Fontanesi, et al., 2004 [7]				
The study used critical path analysis to understand operational factors involved in influenza vaccinations in ambulatory care 16 ambulatory care settings, 666 encounters influenza vaccinations, visits types, OCPE-S used to encode all encounters Timeframe: October 2001–January 2002 Location: USA	Prospective/observational - OCPE-S was used by visit observers to document all activities of the visit and encode all encounters	Visits that resulted in vaccination or did not (missed opportunity visits)	62% of patients were vaccinated; 92 (out of all 243 scheduled) visits were missed opportunity (38% missed opportunity); 56% of visits with incomplete sequence of clinical events did not result in vaccinations; seven clinical factors combined predicted 93% vaccinations	A diagram of best pathway to result in 93% vaccinations and pathways leading to missed opportunity is provided, may be useful in QI and education strategies.
Maurer, et al., 2009 [10]				
Purpose: To investigate the impact of reducing missed opportunities to vaccinate adults against influenza. US adults (*n* = 5067) from a national survey by Knowledge Networks in Menlo Park, CA. Influenza vaccine Timeframe: March 4–7, 2009 Location: USA	Retrospective data analysis of a survey of a national sample of US adults	Missed opportunity calculated as number of unvaccinated patients with at least 1 health care provider visit between October and December 2008 with or without accounting for the patient’s willingness to be vaccinated. Estimated numbers of vaccinated and unvaccinated adults were estimated by scaling up estimated vaccination and missed opportunity rates and disease prevalence.	This method estimates 53 million health care visits between October and December, 2008, but unvaccinated for influenza. Missed opportunity: 14.4% of unvaccinated patients with at least one visit who are amenable to vaccinations. Vaccinating all of those patients would increase overall rate for that time period by 23.1%. Eliminating missed opportunity only among those willing to be vaccinated would result in 14.4% increase.	Limitations: Vague description of methodology. Influenza only. Does not have a detailed description of missed opportunity calculation.

*Abbreviations: VA* Veteran’s Administration; *PPV* pneumococcal polysaccharide vaccine; *USA* United States of America; *UK* United Kingdom; *ER* emergency room; *OCPE-S* Observational Checklist of Patient Encounters-Seniors

### Missed opportunities to immunize operational methodologies

The studies provided variable detail in how the authors defined and determined missed opportunities. Generally, missed opportunity definitions were defined based on data including either visits (encounters), [7, 8] or a combination of patient vaccination status with encounters [9–13] (Table 1).

The most common definition of missed opportunities was estimated retrospectively; chart reviews or surveys were conducted to determine the vaccination status using reports or records of a past visit to a health care facility (Table 1). For example, Murer, et al. estimated missed opportunities based on the proportion of unvaccinated and amenable patients with at least one office visit between October and December, 2008, derived from the retrospective survey data [10].

In one study, missed opportunities were also estimated based on real-time visit observation and reported as a dichotomous outcome of either no vaccine recommendation (missed opportunity) or vaccine administration at the visit (no missed opportunity) [7].

Five of the seven studies did not take into account patients’ willingness or refusal for vaccination when determining missed opportunities [7, 9, 11–13]. Nowalk, et al. incorporated patient refusal into their definition of missed opportunities by reporting visits where the vaccine was not discussed, not given, or not offered and refused by the patient. In that study, missed opportunities defined as above were quantified as the number of visits per patient [8]. Maurer, et al. incorporated patient willingness to be vaccinated in the report [10].

Variations in study design additionally included the length of observation period (from one to 5 years) and the type of healthcare encounter, ranging from a visit to a health care provider, a family physician consult, a hospital outpatient department, an emergency room to hospitalizations.

### Data synthesis and meta-narrative maps of the common methodological approaches in defining missed opportunities

**Fig. 2:**
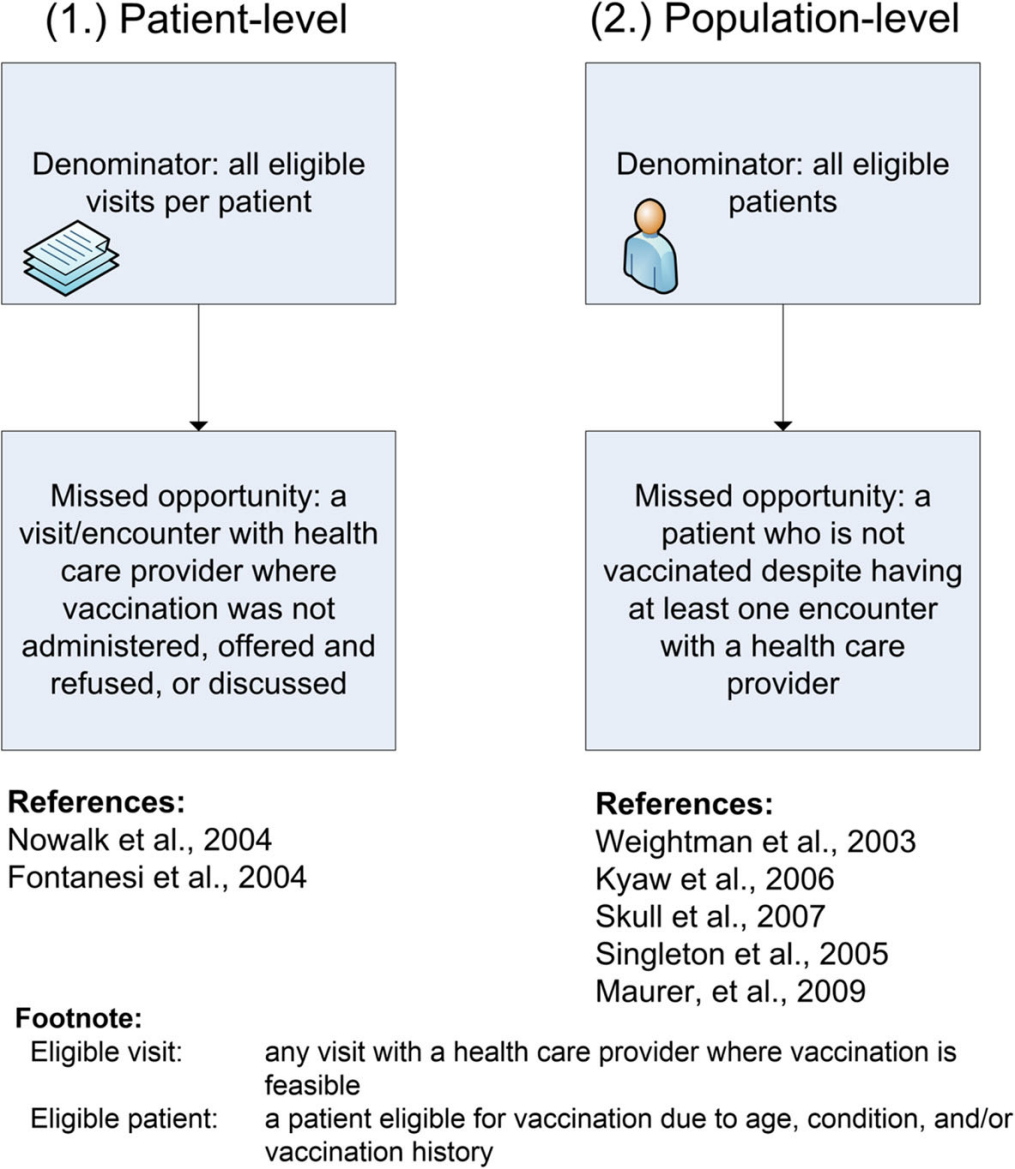
Meta-narrative maps of the common methodological approaches in operationalizing missed opportunities

Here we describe a framework for operationalizing the missed opportunity term based on how the missed opportunity operational methods may be clustered given common methodological characteristics described in the included studies. We synthesized the methodologies identified in the studies and generated the meta-narrative maps of the findings with overall summary of how a “missed opportunity” term may be potentially operationalized (Fig. 2).

Upon data synthesis, the reported methodologies for quantifying missed opportunities fell into two general categories: 1. Based on the number of healthcare encounters without appropriate vaccination services, defined as a number of visits per patient with no vaccination related services (*missed opportunities per patient or Patient-level*) 2. Based on vaccination status as “non-vaccinated” among a group of patients who had a health care encounter where the vaccination should/could have had happened presented as proportion of non-vaccinated patients who had an encounter with health care providers (*missed opportunities per patient population or Population-level*).

From the outcome measure perspective, the studies that used the former patient-level method generated a specific and detailed variable that was characterized by a nominal value per patient presented for a group as a mean and SD, distinctly different form currently used measures of vaccination coverage, i.e., proportion or percentage. The population-level method resulted in a dichotomous variable at the patient level present at the group (population) level as percentage, similar in format to the commonly used vaccination coverage metrics. The population-level missed opportunity measure is, however, distinctly different from the general population metric of vaccine coverage as it assumes the documented health care encounter during a specific time frame, while the vaccination rates do not take in consideration whether the patient actually visited the health care facilities or interacted with the health care.

## Discussion

The results of this study highlighted that *missed opportunity* is variably defined, indirectly assessed if at all, and does not appear to address or connect in a systematic way to what is known about the barriers to immunization. The results of our study, however, provide initial evidence that the missed opportunity term is operational based on the methodologies identified in several existing studies. Moreover, it can be operationalized or quantified as a measurable outcome (variable) using at least two different approaches: based on the results of a visit and based on patient vaccination status.

The diversity of the studies included in this review resulted in two general approaches for *missed opportunity* term operationalization – missed opportunities per patient or Patient-level and missed opportunities per patient population or Population-level. Each approach has their unique strengths and limitations. For example, classifying missed opportunities per patient (patient-level) as in Nowalk study may require higher quality data and documentation, needs to take in account circumstances of every visit, and may be labor-intensive [8]. This approach as such may be better suited for prospective, rigorous quality improvement and research studies with the possibility to track and implement changes in real time.

The data for the missed opportunities for patient population (population-level) seem easier to obtain, and the results are usually presented as percentage. However, that percentage in the studies we reviewed can be as high as 99.8% [12] and as such, a measure derived by this method may be prone to a ceiling effect, can only be calculated retrospectively, may not be a good measure for prospective studies, and is not very actionable because of the retrospective nature. This approach may be more suitable for simple quality improvement studies based on past performance and existing data such as Plan-Do-Study-Act (PDSA-based projects, for example. Overall, given the nascent state of research in this area, apparent lack of missed opportunity term standardization, and a limited number of studies published up to date, it is unclear how representative, accurate or comparable the approaches are based on their performance warranting a need for further research.

Despite the lack of standardization, the data synthesis revealed one common feature among all studies included in this review: that regardless of the methodology used, the missed opportunities are clearly associated with a patient encounter. That makes it easier to operationalize the term and to develop appropriate strategies, given that missed opportunities are directly tied to a patient visit with health care providers who have more control of the visits than of some other barriers to vaccinate.

While that leads to higher expectation placed on providers for stronger vaccine recommendations, [14–16] the promising aspect of addressing missed opportunities at the time when the visit actually occurs is by utilizing the care team-based strategies such as standing orders, registry-based approaches, and other team- and system- level efficacious intervention [17, 18]. Focusing on one visit at a time for appropriate vaccinations for a visiting patient may be a promising quality improvement approach for increasing overall vaccinations rates among health care utilizers pending further testing.

Though investigating the potential causes of missed opportunities is outside of the scope of this study, it is important to mention that one of the reviewed studies provided initial evidence on the possible causes missed opportunities [7]. The authors of that study (Fontanesi, et al.) identified that a substantial proportion of missed opportunities in the study were explained by a combination of several factors with the most influential being an absence of provider-patient immunization discussion during a visit.

Future studies should further explore and summarize the root causes of missed opportunities and barriers related specifically to missed opportunities as they may differ from the overall barriers to vaccinations. Further *missed opportunity* term operationalization will help define the currently blurry differences between “immunization barriers” and missed opportunities. We hypothesize that missed opportunities has the potential to serve as a practice performance measure due to a common characteristic identified in our study, namely, missed opportunities are clearly associated with a patient encounter. When a patient is already at the clinic, the number of applicable barriers is reduced, and yet a visit can still result in missed opportunities even when the actual opportunity, i.e., patient had a contact with healthcare provider, existed. From this perspective, missed opportunities can be viewed as a potential way to reduce barriers related to an actual encounter, such as inconsistent and weak provider recommendations, provider bias, patient resistance and clinic inefficiency.

This study also uncovered some of the areas that the future research studies need to address for successful use of operationalized *missed opportunities* as a measurable outcome (variable) for research and quality improvement projects focused on adult immunizations in primary care.

For example, none of the studies correlated missed opportunities with vaccination rates, were interventional in nature, or provided any normative data or recommendations on how to assess changes in missed opportunities over time. Future investigations addressing lack of standardization around operationalizing and computing missed opportunities could lead to such benefits as saving time and effort by deploying the top-ranked method(s) and design data collection and analysis plans accordingly in research and quality improvement projects. However, in order to compare outcomes of different projects reliably using missed opportunities as an additional vaccination measure and to assess performance and ranking of methods, it is essential to conduct further validation studies of this measure.

Furthermore, it is unclear if vaccination setting is an important contributing factor in the level of missed opportunities as most studies included not only visits to primary care providers but emergency room and hospitalizations, and significant missed opportunities were identified in all settings. Additional variability in how the studies included and defined what constituted a patient visit/encounter needs to be considered in the future as some studies included varying numbers of visits per time period to various settings and encounters where the vaccines were possibly offered but refused by the patient. The studies in this review used various inclusion criteria for what constituted an eligible patient encounter with health care and ranged from scheduled visits with family physicians to hospitalizations. Further standardization of the encounter characteristics will be necessary to improve research and quality improvement methods and accuracy and comparability of results across various efforts. Eligible encounter definition will need to account for several aspects including the type of visits and whether a particular visit is an appropriate opportunity to vaccinate; the type of patients and whether the visiting patient is eligible for certain vaccinations; and the target provider or health care setting and whether the visit can be linked to the provider or the health care site of interest. The development of comparable and validated timeframes for missed opportunities will be useful as the duration of patient encounter observation periods in the absence of rigid vaccination schedules recommended in adults compared to vaccination schedules in children may affect accuracy of estimations in adults.

Furthermore, future studies may need to identify and compare effective interventions and models designed to reduce missed opportunities and ultimately improve vaccination rates among adults.

### Strengths and limitations of present study

Only those studies published between January, 2000, and May, 2015, were included in the literature search; therefore, recent studies in-press at time of review or published later were not included. This may in particular reduce the number of studies included in the review that compute missed opportunities using electronic medical record data and other contemporary approaches. Furthermore, we used the term “missed opportunities” as a keyword for the literature search, but it is possible that - owing to the lexical preferences of authors - other terms are in use with the same intended meaning. We performed an exhaustive, wide-ranging literature search of the PubMed database to identify relevant articles matching an extensive list of search terms (Additional file 1). A follow-on literature review was conducted using author citations and bibliographies of articles that passed our selection criteria. Taken together, we are confident that this approach captured all identifiable articles dealing with missed opportunities in adult immunizations published in the identified time-period.

There are several limitations of the present study that may affect generalizability and validity of results. First, the study focused on adult vaccinations only and as a result may not be generalizable to other patient populations. These may include populations distinct from those included in the study (e.g., children or newborns), or sub-groups within the sample who exhibit distinct characteristics (e.g., elderly adults, immunocompromised patients). Additionally, it is possible that studies utilizing quantifiable missed opportunities as an outcome have been published in other areas relevant to different preventative or clinical services.

Lastly, only a small number of studies remained after applying review inclusion criteria which may limit generalizability or inclusiveness of the classifications proposed. However, the data extracted from that small number of studies allowed us to draw initial conclusions that *missed opportunity* can be operationalized and hold promise as a measurable outcome (variable) for research and quality improvement projects aimed to increase adult immunization recommendation and uptake in primary care. Rather than limitation of the literature search process, these initial results indicate the need for future research in this area.

## Conclusions

Our study provided an initial overview of the methods reported in the literature and concluded that the quantifiable *missed opportunity* holds promise as a measurable outcome (variable) for research and quality improvement projects aimed to increase adult immunization recommendation and uptake in primary care. The preliminary results of our study indicate that the missed opportunities term can be operationalized and point to the need for future studies to test quantifiable missed opportunities as a potentially clinically relevant, actionable, and specific clinical and research outcome.

## Additional file

Additional file 1: Search Strategy. This lists the search strategy used by the librarian to locate papers for this review. (PDF 52 kb)
