# Supplementary material for: Missed opportunities for improving practice performance in adult immunizations: a meta-narrative review of the literature
Source: BMC Fam Pract. 2017 Dec 22;18:108. doi: 10.1186/s12875-017-0694-1 (PMC5741967; doi:10.1186/s12875-017-0694-1)
Supplement: Additional file 1: — Search Strategy. This lists the search strategy used by the librarian to locate papers for this review. (PDF 52 kb) [file 12875_2017_694_MOESM1_ESM.pdf]

Influenza Vaccines/administration & dosage*

- Pneumococcal Vaccines/administration & dosage*
- Vaccination/statistics & numerical data*

Influenza, Human/prevention & control*

Medicare/statistics & numerical data*

Cohort Studies

- Medicare/standards

Pneumococcal Infections/prevention & control*

Health Services/utilization*

Immunization Programs/utilization*

- Opportunistic Infections/epidemiology
- Opportunistic Infections/prevention & control*
- Population Surveillance
- Referral and Consultation/utilization

Reminder Systems*

Primary Health Care/organization & administration

Electronic Health Records*

Medical Records*

“missed opportunities”
